# Supplementary material for: Transient domains of ordered water induced by divalent ions lead to lipid membrane curvature fluctuations
Source: Commun Chem. 2020 Feb 7;3:17. doi: 10.1038/s42004-020-0263-8 (PMC9814626; doi:10.1038/s42004-020-0263-8)
Supplement: Supplementary file 1 — Supplementary Information [file 42004_2020_263_MOESM1_ESM.pdf]

Supplementary Information for "Transient domains of ordered water induced by divalent ions lead to lipid membrane curvature fluctuations"

O. B. Tarun[1], H. I. Okur[1,2], P. Rangamani[3], and S. Roke*[1]

[1]*Laboratory for fundamental BioPhotonics (LBP), Institute of Bioengineering (IBI), and Institute of Materials Science (IMX), School of Engineering (STI), and Lausanne Centre for Ultrafast Science (LACUS), École Polytechnique Fédérale de Lausanne (EPFL), CH-1015 Lausanne, Switzerland*

[2]*Department of Chemistry and National Nanotechnology Research Center (UNAM), Bilkent University, 06800, Ankara, Turkey*

[3]*Department of Mechanical and Aerospace Engineering, University of California, San Diego, La Jolla, CA 92093, USA*

**Corresponding author: sylvie.roke@epfl.ch*

Supplementary Note 1. Spatiotemporal autocorrelation of ion-induced domains

To obtain spatiotemporal dynamics between domains, we applied image correlation spectroscopy originally developed by Petersen et. al[1-6] for laser-scanning systems and extended by Gaborski et. al[7] for uniform illumination. We calculated the normalized spatial autocorrelation function (SACF) and temporal autocorrelation function (TACF) from single frame images (acquisition time of 560 ms, 20 frames total) for each divalent cation in Fig. 1d-f. The full width at half maximum (FWHM) of the SACF reports on the characteristic radius of the domains whereas the FWHM of the TACF reports on the characteristic lifetime of the domains. Supplementary Figure 1a shows the normalized spatial autocorrelation function (SACF). Each data point is the average of 20 frames and solid lines represent fitted Gaussian curves. It can be seen that the average radius of the domains for all three ions is 1.5 microns. The spatial autocorrelation function $g(\xi)$ at ξ = 0 μm, $g(0)$, reports on the density of emitters in the focal volume via $g(0) \sim 1/N$ and N is the density of emitting domains. Supplementary Figure 1a shows that the density of domains is in the order of Ca^{2+} > Ba^{2+} > Mg^{2+}. Supplementary Figure 1b shows the normalized temporal autocorrelation function (TACF) for the three divalent cations. On the time scale of our recording, there is no temporal correlation between the domains. The characteristic time of each domain is, therefore, shorter than the recording time.

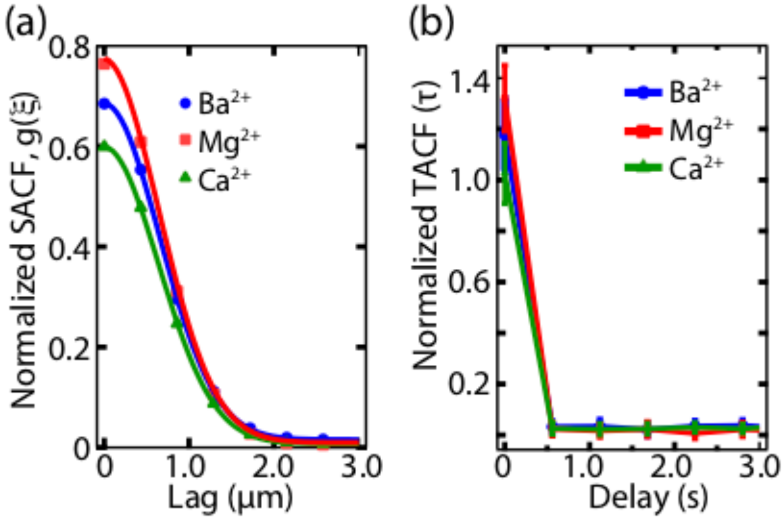

Supplementary Figure 1. Spatiotemporal autocorrelation of ion-induced domains for different divalent cations. (a) Normalized spatial autocorrelation function (SACF) where each data point represent the average of 20 frames (560 ms each) and solid lines are fitted Gaussian curves. **(b)** Normalized temporal autocorrelation function (TACF).

Supplementary Note 2. The intensity distribution of the ion-induced domains

The average intensity distribution of domains in a single frame (560 ms/frame) at different electrolyte conditions is shown in Supplementary Figure 2. The intensity distributions were obtained from N > 1200 domains (distributed over 20 frames). These domains were defined as follows. We used the GDSC ImageJ plugin developed by Alex Herbert[8]. We set threshold intensity (I_{th}) defined as the maximum read-noise plus the maximum dark current noise. This value determines the upper limit of the noise of the system. To determine I_{th} in counts, we calibrated the camera with the gain settings used during acquisition and obtained the number of counts/photo-electron[9]. The average domain size was determined from the SACF of Supplementary Figure 1, and a region of interest represented by a square that has 2 times the FWHM of the SACF as length was used to compute the average domain intensity. The following parameters from the GDSC ImageJ plugin were used: smoothing = 0, box_size = 1, background = 550 (dark current noise), min_height = 305 (read noise upper limit) fraction_above_background = 0, min_width = 0 top_n = 0, neighbour_check border = 2, fit_function=[Free circular], fit_criteria=[Least-squared error] max_iterations = 20, significant_digits = 4, coord_delta = 0.0100, single_fit single_region_size = 5, initial_stddev = 0.000.

Supplementary Figure 2 shows that changing electrolytes increases the average SH response per domain (μ) and spread of values per domain (σ) between the ions in the order Ca^{2+} > Ba^{2+} > Mg^{2+}. Comparing the relative increase between the three ions and removing the SH contribution from the aqueous solution, Ca^{2+} increases the intensity by 163 % compared to Ba^{2+} and 325 % to Mg^{2+}. The spread of the values per domain change by 106 % and 211 %, respectively.

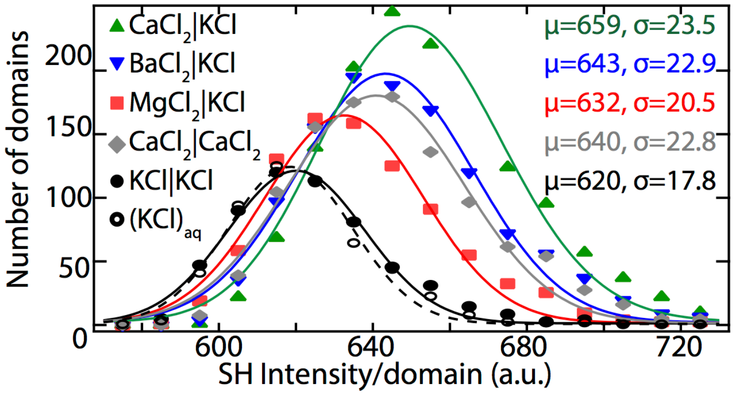

Supplementary Figure 2. Average intensity distribution of domains at different electrolyte conditions. The legend denotes the aqueous solution (I = 150 μM and pH neutral) in contact with a symmetric membrane composed of 70:30 mol % DPhPC:DPhPA. Data points are fitted with a Gaussian distribution and the mean (μ) and standard deviation (σ) are shown.

Supplementary Note 3. Single frame correction for hyper-Rayleigh scattering

Supplementary Figure 3 shows single frame images for Ca^{2+}, Ba^{2+}, and Mg^{2+} interaction membranes. Note that the top row is identical to that of Fig. 2a. These single frame images still contain sparse incoherent SH scattering intensity from the adjacent solution (also known as hyper-Rayleigh scattering, examples of such contribution are indicated in Supplementary Figure 3 with green circles). Supplementary Figure 2 (black curve, open circles) shows the intensity distribution of an electrolyte solution, which arises just from HRS. Since the HRS response is distributed over the image in a random way and since the occurrences are sparse, it is not possible to simply subtract an image (as can be done for longer acquisition times). Therefore, we scaled the intensity distribution per domain using the average occurrence, mean and standard deviations of the HRS intensity recorded from aqueous solution (black curve, open circles in Supplementary Figure 2).

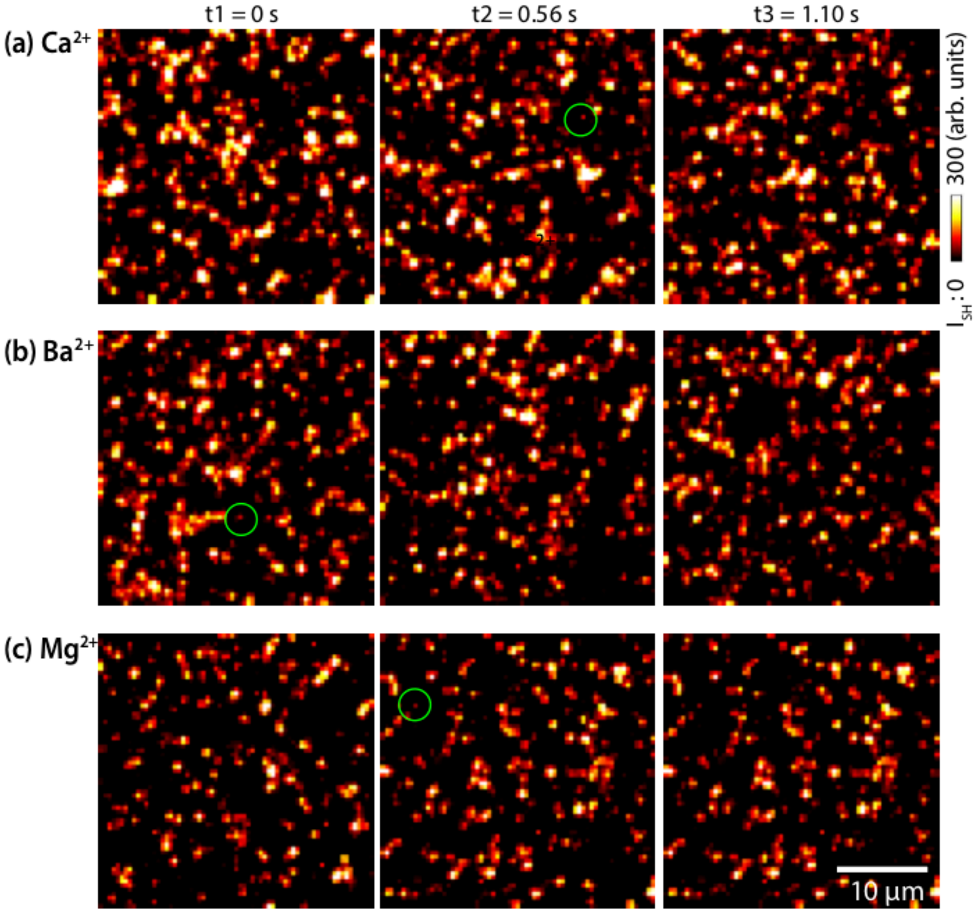

Supplementary Figure 3. Single frame SH images of ion-induced ordered water domains. Time series of SH images (560 ms each) of symmetric membranes composed of 70:30 mol % DPhPC:DPhPA with the bottom leaflet in contact with $(KCl)_{aq}$ and the top leaflet in contact with **(a)** $(CaCl_2)_{aq}$, **(b)** $(BaCl_2)_{aq}$, and **(c)** $(MgCl_2)_{aq}$ at the same ionic strength of 150 µM. All images were collected with all beams P-polarized. The green circles represent examples for potential hyper-Rayleigh contributions.

Supplementary Note 4. From SH intensity to surface potential difference

The SH intensity was converted to surface potential difference as described previously[10,11]. Briefly, following Supplementary Equation 1 (repeated here for convenience):

$$\langle I(2\omega)\rangle_{x,y} = C\,\langle \mathrm{I}(\omega)^2\,\rangle_{x,y}\left|\langle\Delta\chi_s^{(2)}\rangle_{x,y} + f_3\chi^{(3)\prime}\langle\Delta\,\Phi_0\rangle_{x,y}\right|^2 \quad (1)$$

the image averaged SH intensity $\langle I(2\omega)\rangle_{x,y}$ depends on the electrostatic membrane potential $\Delta\Phi_0$. As the electrostatic properties of the image averaged bilayer effectively consists of coupled capacitors, $\Delta\Phi_0$ is connected to an external bias U via $\Delta\Phi_0 = \Delta\Phi_{0,init} + \beta U$. We determined that $\beta = 0.97$. By recording the SH intensity as a function of external bias we determined the unknown values of the constant $C,$ the second-order surface susceptibility difference $\Delta\chi_s^{(2)} = \chi_{s1}^{(2)} - \chi_{s2}^{(2)}$, $\Delta\Phi_{0,init}$, and the effective third-order susceptibility of water $\chi^{(3)\prime}$ for a liquid asymmetric membrane composed of 30:70 mol % negatively charged (with a charge –e per lipid): neutral lipid mixture on one leaflet in contact with a liquid neutral lipid on the other leaflet. Note that $f_3 = 1$. The values determined for $\Delta\chi_s^{(2)}$ and $\chi^{(3)\prime}$ of water were [12]: $\Delta\chi^{(2)} = \chi_{s1}^{(2)} - \chi_{s2}^{(2)}$ ($5 \cdot 10^{-24}$ m^2/V) and $\chi^{(3)\prime}$ =$-10.3 \cdot 10^{-22}$ m^2/V^2. Here, we are dealing with a membrane that is symmetric in composition, with both leaflets composed of 30:70 mol % monovalent negatively charged lipids : neutral lipids. Since this membrane is on average symmetric, we have $\Delta\chi_s^{(2)} = 0$. This means that the calibration curve between membrane potential $\Delta\Phi_0$ and $\langle I(2\omega)\rangle_{x,y}$ will be:

$$\langle I(2\omega)\rangle_{x,y} = C\,\langle \mathrm{I}(\omega)^2\,\rangle_{x,y}\left|\chi^{(3)\prime}\langle\Delta\,\Phi_0\rangle_{x,y}\right|^2 \quad (S2)$$

In absence of asymmetry, $\Delta\Phi_0 \sim U$, and the intensity to membrane potential calibration curve is plotted in Supplementary Figure 4.

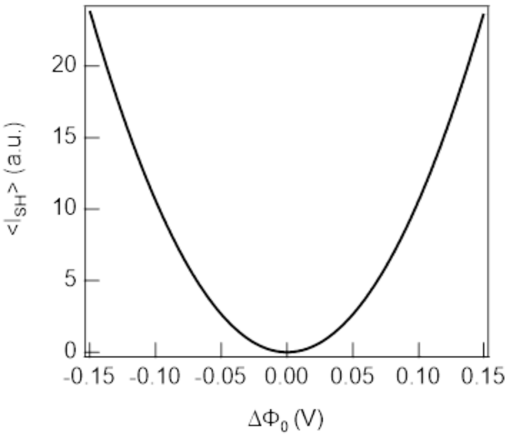

Supplementary Figure 4. Intensity to membrane potential calibration curve.

Supplementary References

1 Petersen, N. O., Höddelius, P. L., Wiseman, P. W., Seger, O. & Magnusson, K. E. Quantitation of membrane receptor distributions by image correlation spectroscopy: concept and application. *Biophysical Journal* **65**, 1135-1146, (1993).

2 Hebert, B., Costantino, S. & Wiseman, P. W. Spatiotemporal Image Correlation Spectroscopy (STICS) Theory, Verification, and Application to Protein Velocity Mapping in Living CHO Cells. *Biophysical Journal* **88**, 3601-3614, (2005).

3 Kolin, D. L., Ronis, D. & Wiseman, P. W. k-Space Image Correlation Spectroscopy: A Method for Accurate Transport Measurements Independent of Fluorophore Photophysics. *Biophysical Journal* **91**, 3061-3075, (2006).

4 Kolin, D. L. & Wiseman, P. W. Advances in Image Correlation Spectroscopy: Measuring Number Densities, Aggregation States, and Dynamics of Fluorescently labeled Macromolecules in Cells. *Cell Biochemistry and Biophysics* **49**, 141-164, (2007).

5 Wiseman, P. W. Image Correlation Spectroscopy: Principles and Applications. *Cold Spring Harbor Protocols* **2015**, pdb.top086124, (2015).

6 Wiseman, P. W. *et al.* Spatial mapping of integrin interactions and dynamics during cell migration by Image Correlation Microscopy. *Journal of Cell Science* **117**, 5521, (2004).

7 Gaborski, T. R., Sealander, M. N., Ehrenberg, M., Waugh, R. E. & McGrath, J. L. Image correlation microscopy for uniform illumination. *Journal of microscopy* **237**, 39-50, (2010).

8 Herbert, A. *Genome Damage and Stability Center (GDSC) Plugin (*http://www.sussex.ac.uk/gdsc/intranet/microscopy/imagej/gdsc_plugins).

9 Li, L., Li, M., Zhang, Z. & Huang, Z.-L. Assessing low-light cameras with photon transfer curve method. *Journal of Innovative Optical Health Sciences* **09**, 1630008, (2016).

10 Tarun, O. B., Hannesschläger, C., Pohl, P. & Roke, S. Label-free and charge-sensitive dynamic imaging of lipid membrane hydration on millisecond time scales. *Proceedings of the National Academy of Sciences* **115**, 4081, (2018).

11 Didier, M. E. P., Tarun, O. B., Jourdain, P., Magistretti, P. & Roke, S. Membrane water for probing neuronal membrane potentials and ionic fluxes at the single cell level. *Nature Communications* **9**, 5287, (2018).

12 Lütgebaucks, C., Gonella, G. & Roke, S. Optical label-free and model-free probe of the surface potential of nanoscale and microscopic objects in aqueous solution. *Physical Review B* **94**, 195410, (2016).
